# Medical Opinion and Movement

**Published:** 1907-08-17

**Authors:** 


### Medical Opinion and Movement.

In an interesting address on "The Progress of the Medical Man" Dr. Frederick Taylor compares the past and present status of the profession, especially from the standpoint of the public, and is led to form an optimistic view of its position and work in the future. Dr. Taylor has many years of zealous work in the profession to look back upon. He has seen several generations of students pass through the gates of Guy's Hospital, and he confidently asserts that not only is the student of the present day required to attain to a vastly higher level of knowledge than did his father or his grandfather, but that he also seems to start at a higher level. Although the medical student in the present day has probably lived down the prejudice which must have been caused in the minds of many people by the picture presented of him by Charles Dickens two generations ago, yet Dr. Taylor thinks there still exists in some minds the idea that the training of the medical student is prejudicial to his moral character, that the practice of dissection and even of surgery blunts his finer feelings. It is only right that any such ideas which may still find expression should be controverted, as they naturally tend to cause a mistrust where absolute confidence is so essential. As Dr. Taylor points out, the students' life in the wards of the hospital teaches them, by contact with the sick and from the example of their seniors and others around them, what is really the lesson of their life's occupation—"That sympathy for which the sick entreat you almost as much as they beg for healing drugs or appliances."

For some time now it has been held by most of the leading neurologists that general paralysis of the insane and locomotor ataxia were different types of the same disease, and were of syphilitic origin, either congenital or acquired. Drs. W. Ford Robertson and Douglas McRae have, however, recently carried out some extensive researches by which they seek to prove that these diseases owe their origin to bacilli of the diphtheroid type, which they are able to distinguish from other similar micro-organisms by their reactions with litmus broth containing one or other of various carbohydrates. Their paper gave rise to a lengthy discussion at a recent meeting of the Medico-Psychological Association, and the general opinion was that the causation of the disease by the bacilli in question was at any rate for the present "not proven." It was pointed out by Dr. Eyre and others that diphtheroid bacilli are widely distributed, are among the commonest organisms found to contaminate pathological material, and are often found in diseases which have no relationship with general paralysis. On the other hand, the authors claim to have produced lesions in rats closely similar to those of general paralysis by feeding them for some time with cultures of these diphtheroid organisms, and they are at present endeavouring to prepare a specific serum for therapeutic purposes. The question must for the present be considered *sub judice*, but if the authors can produce a serum capable of combating these diseases, they will be entitled to regard their diphtheroid bacilli as specific.

What is orthodiagraphy? We venture to think that there are many medical practitioners among those who have not given any special attention to the study of *x*-rays who would be unable to answer this question. Orthodiagraphy, then, is the examination of an object by means of a parallel projection of *x*-rays, and is especially used to determine accurately the outlines of the heart. In the ordinary way the rays emanating from the anticathode of a vacuum tube are divergent. Consequently in ordinary radiographs the picture is magnified, and this is especially so in the case of the heart, as its greatest circumference lies some 7 to 10 cm. away from the surface of the chest. In order, therefore, to obtain an accurate picture of the organ, a parallel projection of the rays must be obtained, and this constitutes orthodiagraphy. The method has been developed chiefly by Professor Moritz, and Dr. Paul C. Franze, of Bad Nauheim, has lately given a demonstration of it in this country. By an adjustable apparatus of bars a pencil is fixed exactly opposite to the centre of the anticathode of the tube, so that a perpendicular ray falls upon the pencil. The point of the pencil projects through the centre of a screen, and the patient is placed between the tube and the screen, with his back towards the tube. By means of the apparatus referred to the tube and screen with the pencil can be carried round the circumference of the heart, and its outline in this way accurately marked out.

Although the Government ship is seriously overloaded, it is to be hoped that the Public Health Bill will not be one of the victims to be thrown overboard. With this Bill Mr. Burns is making an attempt to deal with the question of the proper supervision and inspection of the food supply. Powers are sought to enable the Local Government Board to issue regulations to sanitary authorities in regard to the inspection of food, the destruction of food unfit for consumption, and the provision of clean and decent places for the storage and preparation of food. At present the Board has no power to seize unfit food unless it is exposed for sale, and at only two ports, London and Manchester, have the sanitary authorities power to deal at all effectively with food imported into this country. Mr. Burns drew a lurid picture of the many abominations prevalent in the food traffic, which he hoped to control by the powers he was asking for. But, as Sir Walter Foster pointed out, it is one thing to make regulations and another to enforce their being carried into effect. The local authorities have failed to exercise the powers they already possess, and at present there is no proper machinery to carry out supervision and inspection. In order to give practical effect to the intentions of the Bill there must be an adequate staff of inspectors at the command either of the local authority or the Board, but this practical side of the question does not appear to have received much consideration. In any case we must welcome any attempt to deal with this most important question of ensuring a healthy food supply to the community.

